# High-Grade Pleomorphic Sarcomas Treated with Immune Checkpoint Blockade: The MD Anderson Cancer Center Experience

**DOI:** 10.3390/cancers16091763

**Published:** 2024-05-01

**Authors:** Lewis F. Nasr, Marianne Zoghbi, Rossana Lazcano, Michael Nakazawa, Andrew J. Bishop, Ahsan Farooqi, Devarati Mitra, Beverly Ashleigh Guadagnolo, Robert Benjamin, Shreyaskumar Patel, Vinod Ravi, Dejka M. Araujo, Andrew Livingston, Maria A. Zarzour, Anthony P. Conley, Ravin Ratan, Neeta Somaiah, Alexander J. Lazar, Christina Roland, Emily Z. Keung, Elise F. Nassif Haddad

**Affiliations:** 1 Department of Leukemia, University of Texas MD Anderson Cancer Center, Houston, TX 77030, USA; lfnasr@mdanderson.org (L.F.N.);; 2 Department of Translational Molecular Pathology, University of Texas MD Anderson Cancer Center, Houston, TX 77030, USA; 3 Department of Sarcoma Medical Oncology, University of Texas MD Anderson Cancer Center, Houston, TX 77030, USArbenjami@mdanderson.org (R.B.); maflores@mdanderson.org (M.A.Z.);; 4 Department of Radiation Oncology, University of Texas MD Anderson Cancer Center, Houston, TX 77030, USA; abishop2@mdanderson.org (A.J.B.); afarooqi@mdanderson.org (A.F.);; 5 Department of Genomic Medicine, University of Texas MD Anderson Cancer Center, Houston, TX 77030, USA; 6 Department of Surgical Oncology, University of Texas MD Anderson Cancer Center, Houston, TX 77030, USA; clroland@mdanderson.org (C.R.);; 7 Department of Investigational Cancer Therapeutics, University of Texas MD Anderson Cancer Center, Houston, TX 77030, USA

**Keywords:** immunotherapy, anti-PD1, real world, sarcoma, undifferentiated pleomorphic sarcomas, survival, clinical

## Abstract

**Simple Summary:**

Undifferentiated pleomorphic sarcomas (UPSs) represent 10–20% of all soft tissue sarcomas (STSs) and have quickly emerged as one of the more immune-sensitive types. There are few real-world data on the use of immune checkpoint blockade (ICB) in UPS patients and those with other high-grade pleomorphic STSs. This is a retrospective, observational study of all patients with metastatic high-grade pleomorphic sarcomas treated with FDA-approved ICB at MD Anderson Cancer Center intended to describe the efficacy and toxicity of ICB in this particular group of patients. We find that our outcomes are comparable to those in the published literature and pose a question regarding the need to further evaluate the optimal sequencing of radiotherapy and prior lines of systemic therapy.

**Abstract:**

Background: Undifferentiated pleomorphic sarcomas (UPSs) are amongst the most common subtypes of soft-tissue sarcomas. Few real-world data on the use of immune checkpoint blockade (ICB) in UPS patients and other high-grade pleomorphic STS patients are available. Purpose: The purpose of our study is to describe the efficacy and toxicity of ICB in patients with advanced UPSs and other high-grade pleomorphic sarcomas treated at our institution. Methods: This is a retrospective, observational study of all patients with metastatic high-grade pleomorphic sarcomas treated with FDA-approved ICB at MD Anderson Cancer Center between 1 January 2015 and 1 January 2023. Patients included in trials for which results are not yet published were excluded. Results: Thirty-six patients with advanced/metastatic pleomorphic sarcomas were included. The median age was 52 years. A total of 26 patients (72%) had UPSs and 10 patients (28%) had other high-grade pleomorphic sarcomas. The median follow-up time was 8.8 months. The median PFS was 2.9 months. The 3-month PFS and 6-month PFS were 46% and 32%, respectively. The median OS was 12.9 months. The 12-month OS and 24-month OS were 53% and 29%, respectively. The best response, previous RT, and type of ICB treatment were significantly and independently associated with shorter PFS (*p* = 0.0012, *p* = 0.0019 and *p* = 0.036, respectively). No new safety signal was identified, and the toxicity was overall manageable with no toxic deaths and only four patients (11%) stopping treatment due to toxicity. Conclusions: Real-world retrospective data are consistent with the published literature, with a promising 6-month PFS of 32%. Partial or stable responders to ICB treatment have significantly improved PFS compared to progressors.

## 1. Introduction

Undifferentiated pleomorphic sarcomas (UPSs) are amongst the most common subtypes of soft-tissue sarcomas (STSs), representing 10–20% of all STSs [1,2]. Morphologically, UPS consists of pleomorphic spindle cells with no specific line of differentiation [3] and is a specific entity distinct from other high-grade pleomorphic sarcomas not otherwise specified [4,5]. The mainstay of advanced/metastatic treatment for STSs in general and UPSs in particular consists of systemic therapies, with doxorubicin-based [6,7] and gemcitabine-based treatments used as front-line treatment, with a median progression-free survival (PFS) of around 6 months and a median overall survival (OS) of around 18–20 months [2,8,9,10,11,12].

UPS has quickly emerged as one of the more immune-sensitive types of STS, which was identified during the first immune checkpoint blockade (ICB) clinical trials in patients with advanced and pretreated STS with an objective response rate (ORR) of 20–40% and a median PFS of 3 months [13,14,15,16,17,18,19], whereas other types of sarcomas such as leiomyosarcomas or synovial sarcomas have an ORR < 10% with ICB. Building upon this, several clinical trials have looked at the role of combination therapies with ICB across several STS types, including UPSs, and demonstrated increased efficacy of combination treatments over single agents [20,21,22,23]. These combinations are now moving to the earlier setting including first-line advanced [19] and peri-operative settings [24].

While there is an increasing number of ICB-based clinical trials with UPS patients, few real-world data on the use of ICB in UPS patients and other high-grade pleomorphic STS patients are available. Thus, our aim was to describe the efficacy and toxicity of ICB in patients with advanced UPSs and other high-grade pleomorphic sarcomas treated at our institution.

## 2. Materials and Methods

### 2.1. Study Design

This is a retrospective, observational study of all patients with high-grade pleomorphic sarcomas treated at MD Anderson Cancer Center between 1 January 2015 and 1 January 2023 with anti-PDL1 and anti-CTLA4 ICB. Patients were identified through our MD Anderson Cancer Center’s pharmacy database using the following molecule names: ipilimumab, nivolumab, durvalumab, tremelimumab, atezolizumab, and pembrolizumab. Patients receiving ICB outside of our institution were not identified and, thus, not included. Patients with at least one month of follow-up after initiation of ICB were included. To keep a homogeneous cohort, patients with localized disease receiving ICB treatment in the neoadjuvant setting were excluded. Patients included in trials for which results are not yet published were excluded.

Clinical variables recorded included demographic characteristics such as sex, age, BMI, race and ethnicity, and European Cooperative Oncology Group (ECOG) performance status at initiation of ICB treatment. Disease-associated variables collected included the site of the primary tumor, stage at ICB treatment (locally recurrent/advanced or metastatic), location of metastasis if present, tumor size (biggest dimension, evaluated per RECIST criteria) [25] at ICB, and histologic type. Prior treatment modalities were recorded, including systemic therapies, radiation therapy (RT), and surgical resections. For patients with multiple surgical resections, treatment modalities (chemotherapy regimens and RT) between each surgical resection and any eventual recurrence were recorded. Variables pertaining to ICB treatment included the type of ICB treatment, whether it was administered as a standalone or in combination with another type of systemic therapy or RT, the best radiographic response by Response Evaluation Criteria In Solid Tumors version 1.1 (RECIST1.1) [25], any toxicities experienced, time on treatment, time to progression, and the reason for treatment discontinuation. The last known status of each patient was censored as of 5 April 2023.

Pathology was evaluated by experienced pathologists (RN and AL) in soft-tissue tumors to differentiate between UPSs and other high-grade pleomorphic sarcomas.

ORR is defined as the percentage of patients who achieve a response, whether complete response (complete disappearance of lesions) or partial response (reduction in the sum of maximal tumor diameters by at least 30% or more) per RECIST1.1. Clinical benefit rate (CBR) is defined as the percentage of advanced cancer patients who achieve complete response, partial response, or stable disease for at least 6 months as a result of therapy [25].

This retrospective study of patients treated with sarcomas was approved by the Institutional Review Board.

### 2.2. Statistical Considerations

Categorical variables were reported as percentages and continuous variables as medians and interquartile ranges (IQRs). Comparisons between categorical variables were conducted using Fisher’s exact test. Progression-free survival (PFS) was defined as the time from initiation of ICB treatment to radiographic or clinical progression, death of any cause, or last follow-up, whichever occurred first. Overall survival (OS) was defined as the time from initiation of ICB treatment to death of any cause or last follow-up. The median PFS and OS were calculated using the Kaplan–Meier method, and a 95% confidence interval (95% CI) was estimated. We used the log-rank method to compare the significance of differences between survival curves. The association of clinical factors (disease stage, location of primary, sex, age, ECOG performance status (PS), presence of lung or liver metastasis, number of prior systemic lines, prior receipt of RT, type of ICB received and combination or single agent, and radiographic best response RECIST1.1) with PFS and OS was assessed using Cox univariate and multivariable proportional hazard models. Only those variables that were associated with survival at *p* < 0.1 in univariate models were included in the multivariable model. All analyses were performed using GraphPad Prism version 9 and IBM SPSS version 26.

## 3. Results

### 3.1. Patient Characteristics

Thirty-six patients with advanced/metastatic pleomorphic sarcomas were included in this study. Patient demographics and disease characteristics are outlined in Table 1. The median age of the patients at the time of initiation of ICB treatment was 52 years (range: 22–79), and 66% were male (n = 24/36). Most patients had an ECOG performance status of 1 (n = 23; 64%). The median body mass index (BMI) at the time of ICB treatment was 30.3 (range: 20–52). The disease histology was UPS in 72% of patients (n = 26/36). The median size of the biggest tumor at the start of ICB treatment was 6 cm (range: 1.3–25). The site of the primary tumor was divided between extremities (n = 15; 42%), trunk (n = 15; 42%), and other (n = 6; 16%), respectively. Lung and liver metastases were seen in 72% (n = 26) and 11% (n = 4) of patients, respectively.

Regarding treatment modalities prior to ICB treatment, 61% (n = 22/36) of patients had prior RT, including neoadjuvant or adjuvant RT for primary sarcoma in 36% (n = 8/22) of cases and palliative intent RT in 64% (n = 14/22) of cases. The median time between the last RT treatment and initiation of ICB was 12 months (IQR: 1.9–49.7). Eight patients (22%) received subsequent courses of RT at any time after the start of ICB with a median of two RT treatments (IQR: 1–3). The median number of surgical resections prior to ICB treatment was one (range: 0–8), and the median number of lines of systemic therapy prior to ICB treatment was two (range: 0–10). Patients received prior anthracycline-based and gemcitabine-based chemotherapy in 72% (n = 26/36) and 75% (n = 27/36) of cases, respectively. As 53% (n = 19/36) of patients received their first line of chemotherapy in the peri-operative setting (neoadjuvant and/or adjuvant), the median number of prior systemic lines in the metastatic setting was 1.5 (range 0–6).

The ICB treatments received were atezolizumab (n = 2), durvalumab (n = 11), ipilimumab (n = 2), nivolumab (n = 5), pembrolizumab (n = 14), tremelimumab (n = 11), and other PDL1 inhibitors (n = 3). ICB treatment was given either as single-agent standalone therapy (n = 15; 42%), in combination with another ICB agent (n= 16; 44%), in combination with RT (n= 3; 8%), or in combination with chemotherapy or an antiangiogenic (n = 2; 6%). Twenty-five patients (69%) received ICB as part of a clinical trial.

### 3.2. Responses to ICB

**Figure 1:**
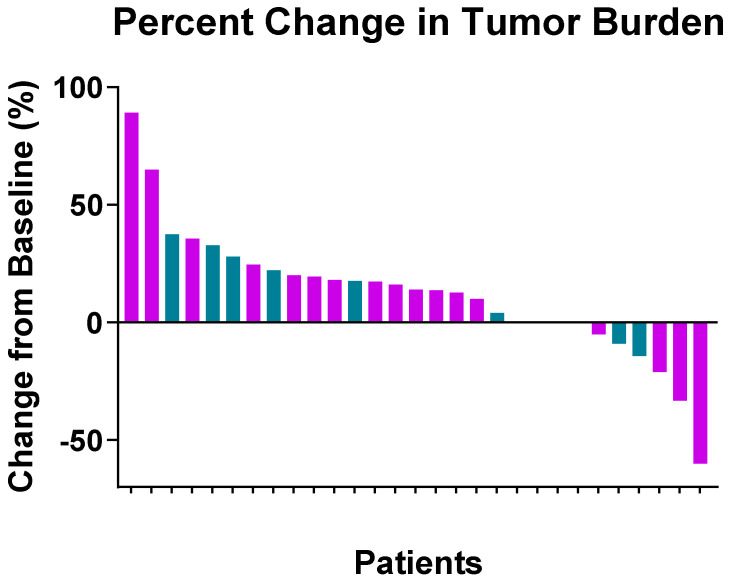
Waterfall plot by histology: purple represents UPS, and blue represents other pleomorphic sarcomas.

The best response of patients to treatment was progression of disease (n = 21; 58%), stable disease (n = 9; 25%), partial response (n = 2; 6%), or complete response (n = 1; 3%). One patient with UPS achieved CR after seven cycles of pembrolizumab, having received prior RT (56 months prior to ICB) and one prior line of gemcitabine-based systemic therapy. Three patients did not have an evaluable response due to logistical issues related to insurance coverage that prevented imaging at MD Anderson after ICB start. Responses are illustrated in the waterfall plot shown in Figure 1.

In the 33 patients with an evaluable response by imaging, the ORR was 9.1% (n = 3/33) and the CBR was 30.3% (n = 10/33). Table 2 shows the response rates according to the studied clinical characteristics. Previous RT, sex, BMI, histology, presence of liver or lung metastasis, number of previous lines of systemic therapy, and type of ICB treatment did not statistically significantly affect CBR. None of the aforementioned characteristics had a significant impact on ORR either.

### 3.3. Progression-Free Survival with ICB

**Figure 2:**
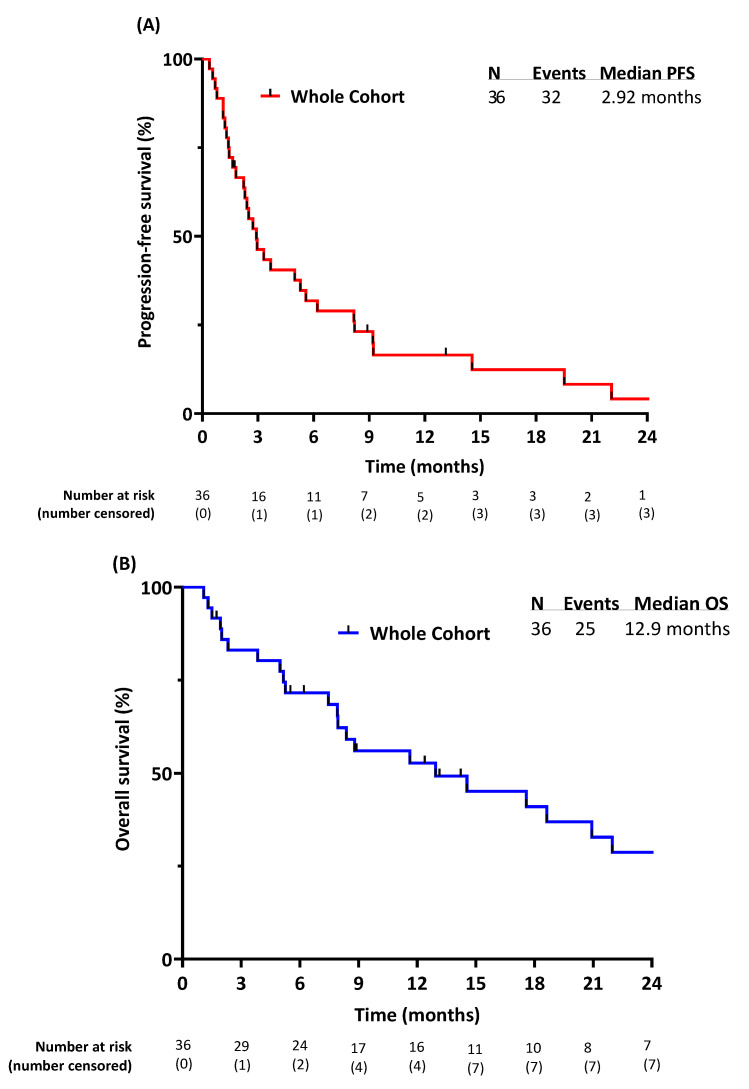
Outcomes of the whole cohort: (**A**) progression-free survival; (**B**) overall survival.

With a median follow-up time of 8.8 months, the median PFS was 2.9 months, as seen in Figure 2A. The 3-month PFS and 6-month PFS were 46% and 32%, respectively.

The median PFS was 2.9 months and 3.8 months in the UPS group and in the other high-grade pleomorphic sarcoma group, respectively.

**Figure 3:**
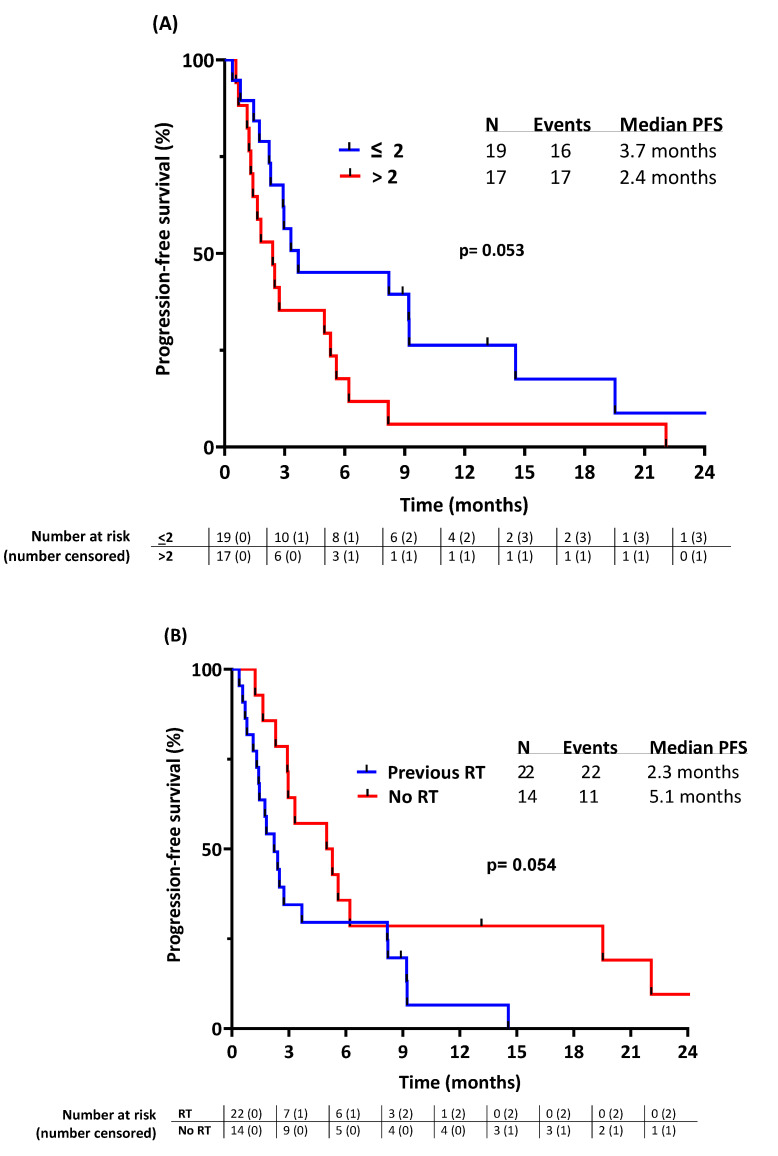

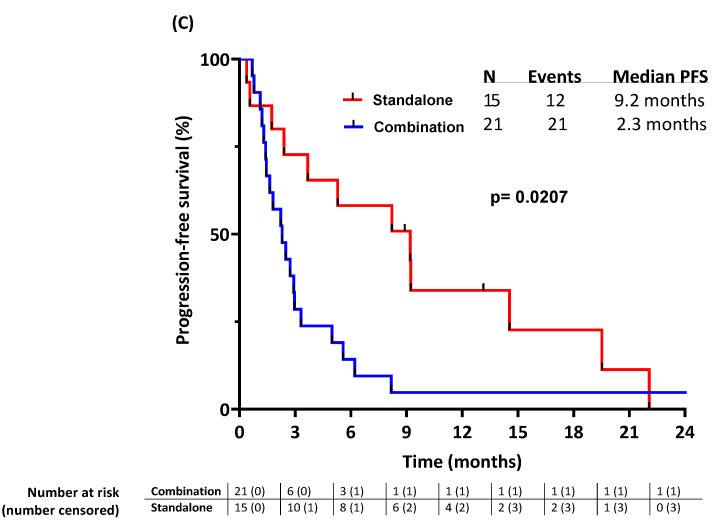
Progression-free survival stratified by (**A**) number of lines of systemic therapy prior to ICB, (**B**) exposure to radiotherapy (RT) prior to ICB, and (**C**) type of ICB treatment.

**Figure 4:**
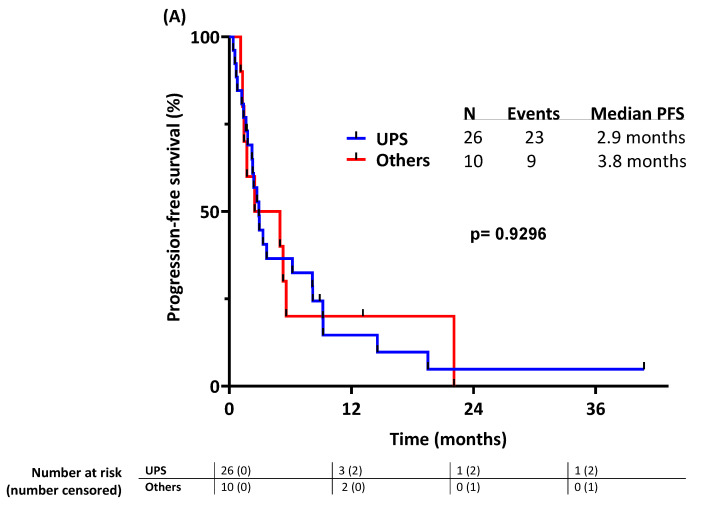

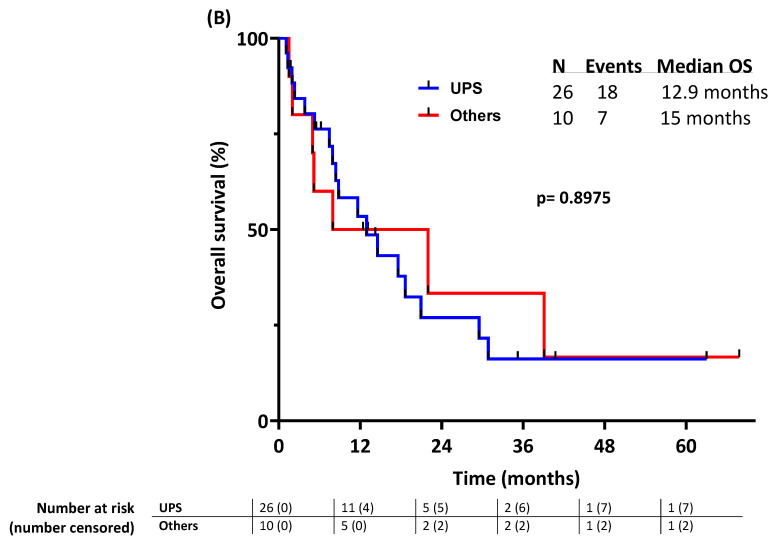
Outcomes stratified by histology: (**A**) progression-free survival; (**B**) overall survival.

In univariate analyses of PFS (Table 3; Figure 3), ICB combination was associated with significantly shorter PFS (combination: 2.3 months vs. no combination 9.2 months, *p* = 0.021) while previous RT, sex, histology, age, presence of liver or lung metastasis, and number of previous lines of systemic therapy did not. Sarcoma histology (UPSs vs. other high-grade pleomorphic sarcomas) did not have a statistically significant impact on PFS (*p* = 0.93) (Figure 4A). The previous number of systemic lines of therapies (>2 vs. 1 or 2) was not significantly associated with PFS despite an HR of 2.06 (*p* = 0.053). Likewise, previous RT was not significantly associated with PFS despite an HR of 0.49 (*p* = 0.054).

**Figure 5:**
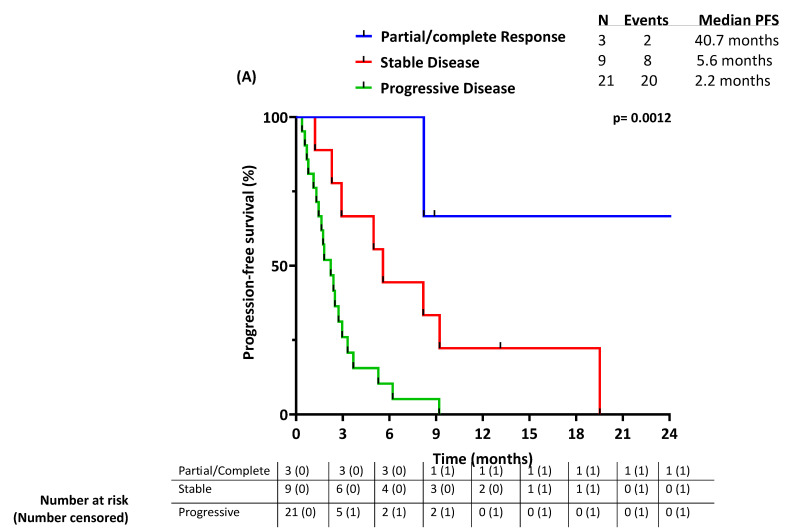

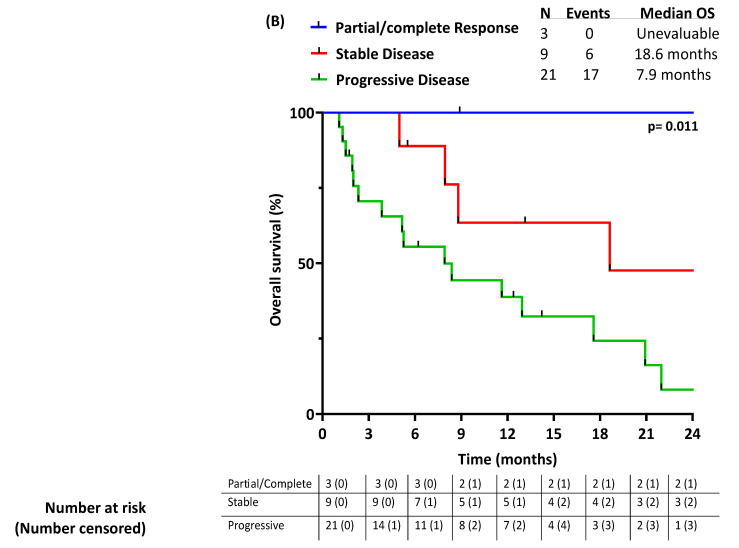
Outcomes stratified by best response to ICB: (**A**) progression-free survival; (**B**) overall survival.

The best response to ICB treatment was significantly associated with PFS (*p* = 0.0012) (Figure 5A): the median PFS was 40.7 months and 5.6 months in patients whose tumors responded (PR/CR) and stabilized (SD) per RECIST 1.1, respectively, compared to 2.2 months in non-responders.

In the multivariate analysis including RT prior to ICB treatment, the number of previous systemic therapies (>2 vs. 1 or 2), and the type of ICB treatment (standalone vs. combination), only previous RT and the type of ICB treatment were significantly and independently associated with shorter PFS (*p* = 0.0019 and *p* = 0.036, respectively, Table 3). There was no significant difference in PFS (*p* = 0.52) between patients receiving peri-operative RT and patients receiving RT with palliative intent in the metastatic setting (Supplementary Figure S1).

### 3.4. Overall Survival with ICB

The median OS was 12.9 months in the whole cohort (Figure 2B). The 12-month OS and 24-month OS rates were 53% and 29%, respectively.

The median OS was 12.9 months and 15 months in the UPS group and in the other high-grade pleomorphic group, respectively.

In the univariate analyses for OS, previous RT had a statistically significant impact on OS (HR = 0.44, previous RT 7.9 months vs. no previous RT 17.5 months, *p* = 0.047, Supplementary Table S1 and Supplementary Figure S2) while age, race and ethnicity, sex, histology, presence of liver or lung metastasis, number of previous lines of systemic therapy (Supplementary Figure S3), and type of ICB combination (Supplementary Figure S4) did not. Sarcoma histology (UPSs vs. other pleomorphic sarcomas) did not have a statistically significant impact on OS (*p* = 0.90) (Figure 4B).

The best response to ICB treatment per RECIST1.1 significantly impacted OS (*p* = 0.011) (Figure 5B): the median OS was not attained and 18.6 months in patients whose tumors responded and stabilized per RECIST 1.1, respectively.

### 3.5. Previous Radiation Therapy

Due to the results on the impact of previous radiation therapy, we performed additional analyses to compare patients who had had previous RT and those who did not. While no significant difference was found between these two groups, patients who had previous RT tended to have larger tumors (biggest diameter 7.7 cm vs. 4 cm; Supplementary Table S2).

Additionally, we performed a subgroup analysis based on the timing of RT prior to ICB. This analysis showed no difference in PFS or OS based on the timing of RT (peri-operative vs. palliative/metastatic setting, Supplementary Figure S1).

### 3.6. ICB Combination

Due to the results on the impact of ICB combination therapy, we performed additional analyses to compare patients who had standalone ICB therapy and those who did not. Patients who had ICB combinations tended to have more prior surgical resections (median of 2 vs. 1 surgical resections for standalone ICB, *p* = 0.013) and more prior lines of therapy (median 3 vs. 1, *p* = 0.062), suggesting that this population was more heavily pretreated. Patients who had ICB combinations also tended to have received ICB as part of a clinical trial (90% of combination ICB vs. 40% of standalone ICB patients; Supplementary Table S3).

### 3.7. Toxicity

No new safety signal was identified, and the toxicity was overall manageable. There were no toxic deaths. Four patients (11%) stopped treatment due to toxicity. Seven patients (19%) experienced > grade 3 toxicities. Five patients (14%) experienced diarrhea or colitis of any grade.

## 4. Discussion

This report aims to describe real-world data on ICB treatment for patients with UPSs and other high-grade pleomorphic STSs. While several prospective clinical trials have identified this subtype of STS as one of the most immune-sensitive types of STS [13,14,26], few real-world histology-specific data exist.

The data reported here are overall consistent with that reported in clinical trials with a median PFS of roughly 3 months for UPS patients [13,14]; however, the ORR was slightly inferior to that previously reported, as ours is around 10% versus 20–30% in clinical trials. Real-world data are often slightly less promising than clinical trials, as patients are usually frailer and have more comorbidities and prior lines of treatment. We also found that ICB in later lines of therapy may be less effective and that RECIST response is associated with PFS with ICB treatment, which is consistent with reports across other cancer types [27,28,29,30,31,32,33].

High-grade pleomorphic sarcomas that do not meet all morphologic criteria for UPSs have often been treated along the same lines as UPSs and are offered ICB in clinical practice, and our report indicates that this approach is reasonable for ICB treatment, given that the 3-month PFS and 6-month PFS were 46% and 32%, respectively. Clinical trials are now including these sarcomas with UPS cohorts testing ICB treatment.

The notable difference in our report compared to clinical trials is that combination ICB seemed inferior to standalone ICB regarding PFS. This is not expected, as multiple trials have shown a benefit of combination ICB compared to single-agent PD1 [21,22,34,35]. However, a potential selection bias could explain this association since clinicians are aware that single-agent PD1 treatment may take longer to be active and can be concerned with the risk of hyperprogressive disease [36]. Thus, in cases of rapidly progressing disease or high tumor burden, clinicians are more likely to try a combination therapy. In contrast, in the case of a slow-growing disease, the added toxicity of a combination therapy is avoided by treating patients with single-agent ICB [37,38]. This selection bias is illustrated by the fact that patients who were treated with combination therapies had a higher number of previous surgical resections and lines of systemic therapies, indicating this population was more heavily pretreated and that there is likely a selection bias in this analysis.

The association between RT and ICB response observed in our study is also likely a result of selection bias. The main hypothesis to explain this is that tumors that recurred and progressed despite RT in the peri-operative setting had intrinsically bad biology and an immune-suppressive microenvironment. Likewise, RT in the metastatic setting would be offered in patients with more symptomatic disease, which may inherently be linked to bad biology. However, there may be biological ties, but the role of previous RT in resistance to ICB is controversial. While concurrent ICB and RT are effective and likely synergistic in the treatment of UPSs and other tumor types, previous RT may have a more immune-suppressive role, as several cytokines, including TGFbeta, and tissue remodeling cells, such as macrophages and neutrophils, are recruited to the tumor after RT [39]. We tried to investigate differences between patients who had previous RT compared to those who did not and did not find any significant differences in our small cohort. Ultimately, our data are hypothesis-generating in nature, and no strong association or mechanistic claim can be made from our clinical report, but the question of RT timing with respect to ICB may be an important one to address in the future. As such, concurrent RT when feasible with ICB is a very promising therapeutic strategy for patients with UPSs.

This study highlights a critical need for the identification of biomarkers of response to ICB for patients with sarcomas. As our cohort is a small, real-world, heterogeneous cohort, we were unable to identify strong predictors of response. This highlights a challenge in deriving data from real-world studies beyond clinical trials but invites further investigation into potential predictors of response.

This is a retrospective, single-center cohort with a small number of patients, and thus, there is significant bias in this analysis, and the statistical power of our findings is limited. The results discussed here are hypothesis-generating and descriptive by nature, and no causality can be inferred.

## 5. Conclusions

Real-world retrospective data demonstrate a median PFS of 2.9 months for patients with UPSs and other high-grade pleomorphic sarcomas treated with ICB in the metastatic setting, which is consistent with the published literature. The optimal sequencing of RT and prior lines of systemic therapy needs to be further evaluated.

## Figures and Tables

**Table 1:** Patient and disease characteristics.

Characteristic	Category	N (%)/Median [Range]		
		Whole Cohort N = 36	UPS N = 26	Other N = 10
Age at first ICB (years)		52 [22–79]	52 [22–79]	53 [30–79]
Sex	Male Female	24 (66) 12 (33)	18 (69)	6 (60)
Race	Caucasian African American	31 (86) 5 (14)	22 (85)	9 (90)
ECOG performance status	0 1 2	11 (30) 23 (64) 2 (6)	10 (38)	1 (10)
BMI		30.3 [20–52]	31 [21–50]	26 [20–52]
Histology	UPS Other unclassified pleomorphic	26 (72) 10 (28)	26 (100) 0	0 10 (100%)
Biggest tumor diameter at start of ICB (cm)		6 [1.3–25]	5.3 [1.3–22]	6.7 [1.5–25]
Lung metastasis		26 (72)	18 (69)	8 (80)
Liver metastasis		4 (11)	2 (8)	2 (20)
Site of primary tumor	Extremities Trunk Other *	15 (42) 15 (42) 6 (16)	12 (46) 10 (38) 4 (16)	3 (30) 5 (50) 2 (20)
Previous RT prior to ICB	Yes	22 (61)	17 (65)	5 (50)
Number of surgical resections prior to ICB		1 [0–8]	1 [0–6]	2 [1–8]
Number of lines of systemic therapy prior to ICB		2 [0–10]	2 [0–5]	4 [1–10]
Type of ICB ** treatment	Standalone ICB Combination of ICB + ICB Combination of ICB + RT Combination of ICB + chemo or antiangiogenic	15 (42) 16 (44) 3 (8) 2 (6)	11 (42) 10 (38) 3 (12) 2 (8)	4 (40) 6 (60) 0 0
Received ICB as part of a clinical trial		25 (69)	18 (69)	7 (70)
Best response to ICB	Partial/complete response Stable disease Progressive disease Unknown	3 (8) 9 (25) 21 (58) 3 (9)	3 (12) 6 (23) 15 (58) 2 (7)	0 3 (30) 6 (60) 1 (10)

* Other sites of disease include abdomen, heart, and lumbar spine, ** ICB drugs used: atezolizumab, durvalumab, ipilimumab, nivolumab, pembrolizumab, tremelimumab. Abbreviations: ICB, immune checkpoint blockade; ECOG, Eastern Cooperative Oncology Group; BMI, Body Mass Index; UPS, undifferentiated pleomorphic sarcoma; RT, radiotherapy.

**Table 2:** Response rates according to clinical characteristics.

Variable N (%)	Number of Patients *	Objective Response Rate (ORR)	ORR *p*-Value	Clinical Benefit Rate (CBR)	CBR *p*-Value
All cohort	33	3 (9.1)	-	10 (30.3)	-
**Histology** UPS Other	24 9	- 3 (12.5) 0 (0)	0.54	- 7 (29.2) 3 (33.3)	1
**Previous** **Radiotherapy** Yes No	20 13	- 2 (10) 1 (7.7)	0.54	- 4 (20) 6 (46)	0.14
**Previous Radiotherapy** **Intent** Peri-op Palliative	12 8	1 (8) 1 (12.5)	1	2 (17) 2 (25)	1
**Number of** **Previous Systemic** **Therapies** ≤2 >2	18 15	- 3 (16.7) 0 (0)	0.233	- 7 (38.9) 3 (20)	0.28
**Lung Metastasis** Yes No	24 9	- 3 (12.5) 0 (0)	0.54	- 9 (37.5) 1 (11.1)	0.22
**Liver Metastasis** Yes No	3 30	- 0 (0) 3 (10)	1	- 0 (0) 9 (30)	0.54
**ICB Combination** **Type** Standalone Combination	13 20	- 2 (15.4) 1 (5)	0.55	- 5 (38.5) 5 (25)	0.46
**Sex** Male Female	21 12	- 2 (9.5) 1 (9.1)	1	- 6 (28.6) 3 (27.3)	1
**BMI** <25 >25	8 25	- 0 (0) 3 (12)	0.56	- 1 (12.5) 9 (36)	0.38

* A total of 33 patients were evaluable for response.

**Table 3:** Progression-free survival according to clinical characteristics.

Variable	Number of Patients	Median PFS (Months, IQR)	Univariate HR (95%CI)	*p*-Value	Cox Multivariate (a) HR (95%CI)	*p*-Value
**Sex** Male Female	- 24 12	- 2.7 (1.6–9) 3.1 (2–5.3)	1.33 (0.60–2.92) - -	0.48 - -		
**Race** Caucasian African American	- 31 5	- 3.3 1.6	0.6 (1.9–2.2) - -	0.4 - -		
**Age** 0–65 >65 years	- 25 11	- 3 (1.5–8.8) 2.5 (1.6–6.6)	1.2 (0.5–2.6) - -	0.68 - -		
**Performance Status** 0, 1 2+	-					
**Histology** UPS Other unclassified pleomorphic	- 26 10	- 2.9 (1.68–7.64) 3.8 (1.64–6.21)	0.97 (0.44–2.09) - -	0.92		
**Previous Radiotherapy** Yes No	- 22 14	- 2.2 (1.3–3.5) 5.4 (3–13.1)	0.49 (0.24–1.01) - -	0.054 - -	**0.39 (0.18–0.86)**	**0.019**
**ICB Combination Type** Standalone Combination	- 15 21	- 9.2 (2.1–11.2) 2.3 (1.4–3.32)	**0.42 (0.23–0.92)** - -	**0.0207** - -	**0.4 (0.17–0.94)**	**0.036**
**Number of Previous Systemic Therapies** ≤2 >2	- 19 17	- 3.68 (1.9–9.2) 2.4 (1.3–5.3)	2.06 (0.93–4.29) - -	0.053 - -	0.65 (0.29–1.45)	0.29
**Lung Metastasis** Yes No	- 26 10	- 3 (1.4–5.6) 2.8 (1.9–8.5)	0.95 (0.45–2.0) -	0.94 - -		
**Liver Metastasis** Yes No	- 4 32	- 2.5 (2–7.6) 3 (1.5–7.2)	1.012 (0.34–3.0) -	0.98 - -		

PFS = progression-free survival; ICB = immune checkpoint blockade; HR = hazard ratio; RT = radiotherapy; UPS = undifferentiated pleomorphic sarcoma; (a) multivariate model included previous radiotherapy, number of previous systemic therapies, and type of ICB combination. Values in bold have *p*-values < 0.05.

## Data Availability

Data will not be made publicly available. Anonymized data will be shared upon reasonable academic request and will be subject to legal data transfer agreements.

## Supplementary Materials

The following supporting information can be downloaded at: https://www.mdpi.com/article/10.3390/cancers16091763/s1, Table S1. Overall Survival according to clinical characteristics; Table S2. Patient and disease characteristics by exposure to RT prior to ICB; Table S3. Patient and disease characteristics by type ICB treatment; Figure S1. Progression-free survival in patients with previous RT comparing peri-op vs metastatic RT; Figure S2. Overall Survival stratified by exposure to RT prior to ICB; Figure S3. Overall Survival stratified by number of lines of systemic therapy prior to ICB; Figure S4. Overall Survival stratified by type of ICB treatment.

## References

[1] Penel N., Coindre J.-M., Giraud A., Terrier P., Ranchere-Vince D., Collin F., Guellec S.L.E., Bazille C., Lae M., de Pinieux G. (2018). Presentation and outcome of frequent and rare sarcoma histologic subtypes: A study of 10,262 patients with localized visceral/soft tissue sarcoma managed in reference centers. Cancer.

[2] Savina M., Le Cesne A., Blay J.-Y., Ray-Coquard I., Mir O., Toulmonde M., Cousin S., Terrier P., Ranchere-Vince D., Meeus P. (2017). Patterns of care and outcomes of patients with METAstatic soft tissue SARComa in a real-life setting: The METASARC observational study. BMC Med..

[3] Kelleher F., Viterbo A. (2013). Histologic and Genetic Advances in Refining the Diagnosis of “Undifferentiated Pleomorphic Sarcoma”. Cancers.

[4] Fletcher C.D. (1992). Pleomorphic malignant fibrous histiocytoma: Fact or fiction? A critical reappraisal based on 159 tumors diagnosed as pleomorphic sarcoma. Am. J. Surg. Pathol..

[5] Fletcher C.D. (2014). The evolving classification of soft tissue tumours—An update based on the new 2013 WHO classification. Histopathology.

[6] Benjamin R.S., Wiernik P.H., Bachur N.R. (1974). Adriamycin chemotherapy—Efficacy, safety, and pharmacologic basis of an intermittent single high-dosage schedule. Cancer.

[7] Gronchi A., Palmerini E., Quagliuolo V., Martin Broto J., Lopez Pousa A., Grignani G., Brunello A., Blay J.Y., Tendero O., Diaz Beveridge R. (2020). Neoadjuvant Chemotherapy in High-Risk Soft Tissue Sarcomas: Final Results of a Randomized Trial From Italian (ISG), Spanish (GEIS), French (FSG), and Polish (PSG) Sarcoma Groups. J. Clin. Oncol..

[8] Seddon B., Strauss S.J., Whelan J., Leahy M., Woll P.J., Cowie F., Rothermundt C., Wood Z., Benson C., Ali N. (2017). Gemcitabine and docetaxel versus doxorubicin as first-line treatment in previously untreated advanced unresectable or metastatic soft-tissue sarcomas (GeDDiS): A randomised controlled phase 3 trial. Lancet Oncol..

[9] Tap W.D., Jones R.L., Van Tine B.A., Chmielowski B., Elias A.D., Adkins D., Agulnik M., Cooney M.M., Livingston M.B., Pennock G. (2016). Olaratumab and doxorubicin versus doxorubicin alone for treatment of soft-tissue sarcoma: An open-label phase 1b and randomised phase 2 trial. Lancet.

[10] Tap W.D., Papai Z., Van Tine B.A., Attia S., Ganjoo K.N., Jones R.L., Schuetze S., Reed D., Chawla S.P., Riedel R.F. (2017). Doxorubicin plus evofosfamide versus doxorubicin alone in locally advanced, unresectable or metastatic soft-tissue sarcoma (TH CR-406/SARC021): An international, multicentre, open-label, randomised phase 3 trial. Lancet Oncol..

[11] Maki R.G., Wathen J.K., Patel S.R., Priebat D.A., Okuno S.H., Samuels B., Fanucchi M., Harmon D.C., Schuetze S.M., Reinke D. (2007). Randomized Phase II Study of Gemcitabine and Docetaxel Compared With Gemcitabine Alone in Patients With Metastatic Soft Tissue Sarcomas: Results of Sarcoma Alliance for Research Through Collaboration Study 002. J. Clin. Oncol..

[12] Kim J.H., Park H.S., Heo S.J., Kim S.K., Han J.W., Shin K.H., Kim S.H., Hur H., Kim K.S., Choi Y.D. (2019). Differences in the Efficacies of Pazopanib and Gemcitabine/Docetaxel as Second-Line Treatments for Metastatic Soft Tissue Sarcoma. Oncology.

[13] Tawbi H.A., Burgess M., Bolejack V., Van Tine B.A., Schuetze S.M., Hu J., D’Angelo S., Attia S., Riedel R.F., Priebat D.A. (2017). Pembrolizumab in advanced soft-tissue sarcoma and bone sarcoma (SARC028): A multicentre, two-cohort, single-arm, open-label, phase 2 trial. Lancet Oncol..

[14] Burgess M.A., Bolejack V., Schuetze S.M., Van Tine B.A., Attia S., Riedel R.F., Hu J., Davis L.E., Okuno S.H., Priebat D.A. (2019). Clinical activity of pembrolizumab (P) in undifferentiated pleomorphic sarcoma (UPS) and dedifferentiated/pleomorphic liposarcoma (LPS): Final results of SARC028 expansion cohorts. J. Clin. Oncol..

[15] Keung E.Z., Burgess M., Salazar R., Parra E.R., Rodrigues-Canales J., Bolejack V., Van Tine B.A., Schuetze S.M., Attia S., Riedel R.F. (2020). Correlative Analyses of the SARC028 Trial Reveal an Association Between Sarcoma-Associated Immune Infiltrate and Response to Pembrolizumab. Clin. Cancer Res..

[16] Petitprez F., de Reynies A., Keung E.Z., Chen T.W., Sun C.M., Calderaro J., Jeng Y.M., Hsiao L.P., Lacroix L., Bougouin A. (2020). B cells are associated with survival and immunotherapy response in sarcoma. Nature.

[17] Kelly C.M., Qin L.X., Whiting K.A., Richards A.L., Avutu V., Chan J.E., Chi P., Dickson M.A., Gounder M.M., Keohan M.L. (2023). A Phase II Study of Epacadostat and Pembrolizumab in Patients with Advanced Sarcoma. Clin. Cancer Res..

[18] Liu J., Fan Z., Bai C., Li S., Xue R., Gao T., Zhang L., Tan Z., Fang Z. (2021). Real-world experience with pembrolizumab in patients with advanced soft tissue sarcoma. Ann. Transl. Med..

[19] Pollack S.M., Redman M.W., Baker K.K., Wagner M.J., Schroeder B.A., Loggers E.T., Trieselmann K., Copeland V.C., Zhang S., Black G. (2020). Assessment of Doxorubicin and Pembrolizumab in Patients With Advanced Anthracycline-Naive Sarcoma: A Phase 1/2 Nonrandomized Clinical Trial. JAMA Oncol..

[20] D’Angelo S.P., Melchiori L., Merchant M.S., Bernstein D., Glod J., Kaplan R., Grupp S., Tap W.D., Chagin K., Binder G.K. (2018). Antitumor Activity Associated with Prolonged Persistence of Adoptively Transferred NY-ESO-1 (c259)T Cells in Synovial Sarcoma. Cancer Discov..

[21] Movva S., Avutu V., Chi P., Dickson M.A., Gounder M.M., Kelly C.M., Keohan M.L., Meyers P.A., Cohen S.M., Hensley M.L. (2023). A pilot study of lenvatinib plus pembrolizumab in patients with advanced sarcoma. J. Clin. Oncol..

[22] Rosenbaum E., Qin L.-X., Dickson M.A., Keohan M.L., Gounder M.M., Chi P., Movva S., Kelly C.M., Avutu V., Chan J.E. (2023). Interim results of a phase II trial of first line retifanlimab (R) plus gemcitabine and docetaxel (GD) in patients (pts) with advanced soft tissue sarcoma (STS). J. Clin. Oncol..

[23] Van Tine B.A., Eulo V., Toeniskoetter J., Ruff T., Luo J., Kemp L., Moreno Tellez C., Weiss M.C., Hirbe A.C., Meyer C.F. (2023). Randomized phase II trial of cabozantinib combined with PD-1 and CTLA-4 inhibition versus cabozantinib in metastatic soft tissue sarcoma. J. Clin. Oncol..

[24] Roland C.L., Nassif Haddad E.F., Keung E.Z., Wang W.L., Lazar A.J., Lin H., Chelvanambi M., Parra E.R., Wani K., Guadagnolo B.A. (2024). A randomized, non-comparative phase 2 study of neoadjuvant immune-checkpoint blockade in retroperitoneal dedifferentiated liposarcoma and extremity/truncal undifferentiated pleomorphic sarcoma. Nat. Cancer.

[25] Eisenhauer E.A., Therasse P., Bogaerts J., Schwartz L.H., Sargent D., Ford R., Dancey J., Arbuck S., Gwyther S., Mooney M. (2009). New response evaluation criteria in solid tumours: Revised RECIST guideline (version 1.1). Eur. J. Cancer.

[26] Keung E.Z.-Y., Nassif E.F., Lin H.Y., Lazar A.J., Torres K.E., Wang W.-L., Guadagnolo B.A., Bishop A.J., Hunt K., Feig B.W. (2022). Randomized phase II study of neoadjuvant checkpoint blockade for surgically resectable undifferentiated pleomorphic sarcoma (UPS) and dedifferentiated liposarcoma (DDLPS): Survival results after 2 years of follow-up and intratumoral B-cell receptor (BCR) correlates. J. Clin. Oncol..

[27] Dall’Olio F.G., Marabelle A., Caramella C., Garcia C., Aldea M., Chaput N., Robert C., Besse B. (2022). Tumour burden and efficacy of immune-checkpoint inhibitors. Nat. Rev. Clin. Oncol..

[28] Chalabi M., Fanchi L.F., Dijkstra K.K., Van den Berg J.G., Aalbers A.G., Sikorska K., Lopez-Yurda M., Grootscholten C., Beets G.L., Snaebjornsson P. (2020). Neoadjuvant immunotherapy leads to pathological responses in MMR-proficient and MMR-deficient early-stage colon cancers. Nat. Med..

[29] Cascone T., William W.N., Jr Weissferdt A., Leung C.H., Lin H.Y., Pataer A., Godoy M.C.B., Carter B.W., Federico L., Reuben A. (2021). Neoadjuvant nivolumab or nivolumab plus ipilimumab in operable non-small cell lung cancer: The phase 2 randomized NEOSTAR trial. Nat. Med..

[30] Amaria R.N., Reddy S.M., Tawbi H.A., Davies M.A., Ross M.I., Glitza I.C., Cormier J.N., Lewis C., Hwu W.J., Hanna E. (2018). Neoadjuvant immune checkpoint blockade in high-risk resectable melanoma. Nat. Med..

[31] Gross N.D., Miller D.M., Khushalani N.I., Divi V., Ruiz E.S., Lipson E.J., Meier F., Su Y.B., Swiecicki P.L., Atlas J. (2022). Neoadjuvant Cemiplimab for Stage II to IV Cutaneous Squamous-Cell Carcinoma. N. Engl. J. Med..

[32] Klemen N.D., Hwang S., Bradic M., Rosenbaum E., Dickson M.A., Gounder M.M., Kelly C.M., Keohan M.L., Movva S., Thornton K.A. (2021). Long-term Follow-up and Patterns of Response, Progression, and Hyperprogression in Patients after PD-1 Blockade in Advanced Sarcoma. Clin. Cancer Res..

[33] Nishino M., Ramaiya N.H., Hatabu H., Hodi F.S. (2017). Monitoring immune-checkpoint blockade: Response evaluation and biomarker development. Nat. Rev. Clin. Oncol..

[34] Somaiah N., Conley A.P., Parra E.R., Lin H., Amini B., Solis Soto L., Salazar R., Barreto C., Chen H., Gite S. (2022). Durvalumab plus tremelimumab in advanced or metastatic soft tissue and bone sarcomas: A single-centre phase 2 trial. Lancet Oncol..

[35] D’Angelo S.P., Mahoney M.R., Van Tine B.A., Atkins J., Milhem M.M., Jahagirdar B.N., Antonescu C.R., Horvath E., Tap W.D., Schwartz G.K. (2018). Nivolumab with or without ipilimumab treatment for metastatic sarcoma (Alliance A091401): Two open-label, non-comparative, randomised, phase 2 trials. Lancet Oncol..

[36] Champiat S., Ferrara R., Massard C., Besse B., Marabelle A., Soria J.C., Ferte C. (2018). Hyperprogressive disease: Recognizing a novel pattern to improve patient management. Nat. Rev. Clin. Oncol..

[37] Morad G., Helmink B.A., Sharma P., Wargo J.A. (2021). Hallmarks of response, resistance, and toxicity to immune checkpoint blockade. Cell.

[38] Sharma P., Allison J.P. (2015). The future of immune checkpoint therapy. Science.

[39] Rodriguez-Ruiz M.E., Vitale I., Harrington K.J., Melero I., Galluzzi L. (2020). Immunological impact of cell death signaling driven by radiation on the tumor microenvironment. Nat. Immunol..

