# Supplementary material for: High-Grade Pleomorphic Sarcomas Treated with Immune Checkpoint Blockade: The MD Anderson Cancer Center Experience
Source: Cancers (Basel). 2024 May 1;16(9):1763. doi: 10.3390/cancers16091763 (PMC11083765; doi:10.3390/cancers16091763)
Supplement: Supplementary file 1 [file cancers-16-01763-s001.zip › cancers-2948488-supplementary.pdf]

Title: Undifferentiated Sarcomas Treated with Immune Checkpoint Blockade: The MD Anderson Cancer Center Experience

Supplemental Table S1. Overall Survival according to clinical characteristics.

Supplemental Table S2. Patient and disease characteristics by exposure to RT prior to ICB

Supplemental Table S3. Patient and disease characteristics by type ICB treatment

Supplemental Figure S1. Progression-free survival in patients with previous RT comparing peri-op vs metastatic RT

Supplemental Figure S2. Overall Survival stratified by exposure to RT prior to ICB

Supplemental Figure S3. Overall Survival stratified by number of lines of systemic therapy prior to ICB

Supplemental Figure S4. Overall Survival stratified by type of ICB treatment

Supplemental Table S1. Overall Survival according to clinical characteristics.

Variable	Number of Patients	Median OS (months, IQR)	Univariate HR (95%CI)	p-Value
**Sex**	-	-	1.04 (0.43-2.55)	0.93
Male	24	14.6 (1.1-67.8)	-	-
Female	12	12.9 (5-35.2)	-	-
**Race**	-	-	0.25 (0.05-1.2)	0.08
Caucasian	31	14.6 (1.3-67.8)	-	-
African American	5	5.3 (1.1-13.1)	-	-
**Age**	-	-	1.4 (0.6-3.4)	0.92
0-65	25	12.9 (5.5-17.6)	-	-
>65 years	11	18.6 (3.5-20.3)	-	-
**Histology**	-	-	0.95 (0.4-2.25)	0.90
UPS	26	8.9 (5.3-18.4)	-	
Other unclassified pleomorphic	10	10.2 (5-19.8)	-	
**Previous Radiotherapy**	-	-	**0.44 (0.2-0.99)**	**0.047**
Yes	22	7.9 (2.1-14.5)	-	-
No	14	17.5 (8.1-34.4)	-	-
**ICB Combination Type**	-	-	0.5 (0.23-1.12)	0.094
Standalone	15	13.1 (3.6-32.3)	-	-
Combination	21	8 (5.2-17.6)	-	-
				-
**Number of previous Systemic Therapies**	-	-	0.78 (0.35-1.7)	0.53
≤2	19	12.9 (5-18.1)	-	-
>2	17	7.5 (5.2-20.9)	-	-
**Lung Metastasis**	-	-	1.02 (0.4-2.59)	0.96
Yes	26	12 (5.7-20.4)		-

No	10	5.8 (4.2-12.9)	-	-
**Liver Metastasis**	-	-	0.88 (0.24-3.1)	0.84
Yes	4	6.5 (4.8-22.9)	-	-
No	32	10.3 (5.2-19.2)		-

OS = overall survival; ICB= Immune Checkpoint Blockade; HR = hazard ratio; RT = Radiotherapy; UPS = undifferentiated pleomorphic sarcoma; Values in bold have p-value < 0.05

Supplemental Table S2. Patient and disease characteristics stratified by exposure to RT prior to ICB

Characteristic	Category	N (%) / median [range]		
		**Previous RT N= 22**	**No prior RT N=14**	**p-value**
Age at first ICB (years)	All	53 [22-79]	52 [33-78]	0.8
	>65 years	6 (27)	5 (36)	
Sex	Male	17 (77)	7 (50)	0.2
	Female	5 (23)	7 (50)	
Race	Caucasian	19 (86)	12 (86)	0.9
	African American	3 (14)	2 (14)	
ECOG performance status	0	5 (23)	6 (43)	0.3
	1	15 (68)	8 (57)	
	2	2 (9)	0 (0)	
BMI		32 [20-50]	28 [24-52]	0.3
Histology	UPS	17 (77)	9 (64)	0.6
	Other unclassified pleomorphic	5 (23)	5 (36)	
Biggest tumor diameter at start of ICB (cm)		7.7 [1.3,25]	4 [1.5,14.2]	**0.027**
Lung metastasis		16 (73)	10 (71)	1
Liver metastasis		3 (14)	1 (7)	1
Site of primary tumor	Extremities	9 (41)	6 (43)	
	Chest	10 (45)	5 (36)	
	Other*	3 (14)	3 (21)	
Number of surgical resections prior to ICB		2 [0-8]	2 [0-4]	0.3
Number of lines of systemic therapy prior to ICB		2 [0-10]	3 [0-5]	0.3
Type of ICB** treatment	Standalone ICB	11 (50)	4 (29)	0.11
	Combination of ICB + ICB	10 (45)	6 (43)	
	Combination of ICB + RT	0 (0)	3 (21)	
	Combination of ICB + chemo or antiangiogenic	1 (5)	1 (7)	
Received ICB as part of a clinical trial		14 (64)	11 (79)	0.6
Best response to ICB	Partial/complete Response	2 (9)	1 (7)	0.074
	Stable Disease	2 (9)	7 (50)	
		16 (73)		

Progressive		5 (36)
Disease	2 (9)	
Unknown		1 (7)

*Other sites of disease include abdomen, heart, and lumbar spine
** ICB drugs used: atezolizumab, durvalumab, ipilimumab, nivolumab, pembrolizumab, tremelimumab

Abbreviations: ICB, immune checkpoint blockade; ECOG, Eastern Cooperative Oncology Group; BMI, Body Mass Index; UPS, undifferentiated pleomorphic sarcoma; RT, radiotherapy

Supplemental Table S3. Patient and disease characteristics by type ICB treatment

Characteristic	Category	N (%) / median [range] Standalone ICB N= 15	Combination N=21	p-value
Age at first ICB (years)	All	57 [36, 79]	51 [22, 78]	0.064
Sex	Female	3 (20)	9 (43)	0.3
	Male	12 (80)	12 (57)	
Race	African American	4 (27)	1 (5)	0.2
	Caucasian	11 (73)	20 (95)	
ECOG performance status	0	4 (27)	7 (33)	0.2
	1	9 (60)	14 (67)	
	2	2 (13)	0 (0)	
BMI		30 [24, 52]	31 [20, 44]	0.2
Histology	UPS	11 (73)	15 (71)	1
	Other unclassified pleomorphic	4 (27)	6 (29)	
Biggest tumor diameter at start of ICB (cm)		9.5 [1.5, 22.0]	5.0 [1.3, 25.0]	0.2
Lung metastasis		10 (67)	16 (76)	0.8
Liver metastasis		1 (7)	3 (14)	0.9
Site of primary tumor	Chest	5 (33)	10 (48)	0.7
	Extremities	7 (47)	8 (38)	
	Other*	3 (20)	3 (14)	
Number of surgical resections prior to ICB		1 [0, 3]	2 [0, 8]	**0.013**
Number of lines of systemic therapy prior to ICB		2 [0, 5]	3 [0, 10]	0.062
Previous RT prior to ICB		11 (73)	11 (52)	0.4
Received ICB as part of a clinical trial		6 (40)	19 (90)	**0.004**
Best response to ICB	Partial/complete Response	2 (14)	1 (5)	0.6
	Stable Disease	3 (20)	13 (61)	
	Progressive Disease	8 (53)	6 (29)	
	Unknown	2 (13)	1 (5)	

Abbreviations: ICB, immune checkpoint blockade; ECOG, Eastern Cooperative Oncology Group; BMI, Body Mass Index; UPS, undifferentiated pleomorphic sarcoma; RT, radiotherapy

* Other sites of disease include abdomen, heart, and lumbar spine

Supplemental Figure S1. Progression-free survival in patients with previous RT comparing peri-op vs metastatic RT

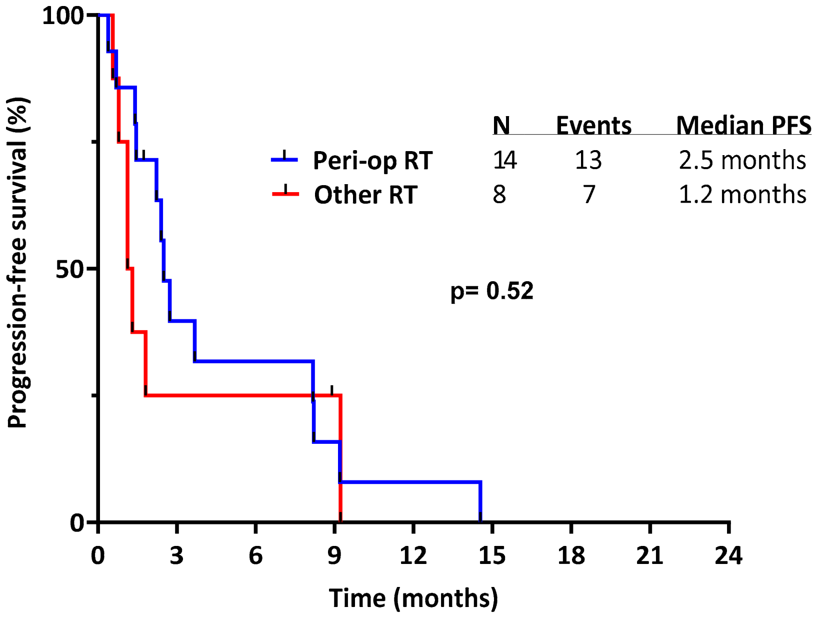

Number at risk (number censored)										
	Peri-op RT	14 (0)	5 (1)	4 (1)	2 (1)	1 (1)	0 (1)	0 (1)	0 (1)	0 (1)
	Other RT	8 (0)	2 (0)	2 (0)	1 (1)	0 (1)	0 (1)	0 (1)	0 (1)	0 (1)

Supplemental Figure S2. Overall Survival stratified by exposure to RT prior to ICB

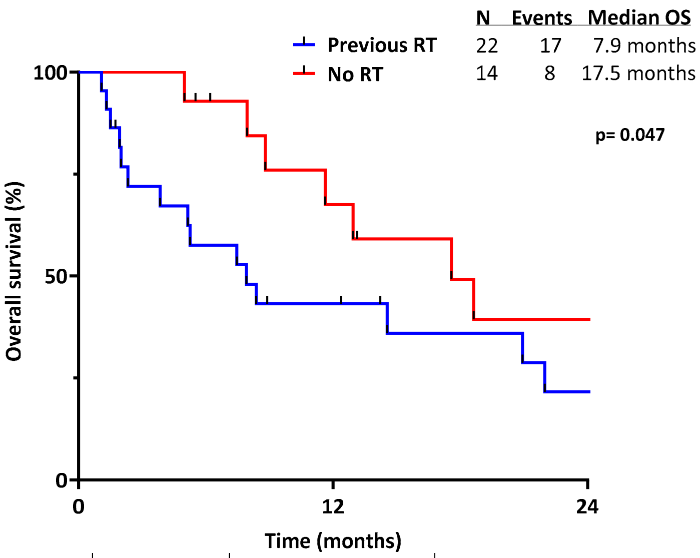

Number at risk (number censored)				
	Previous RT	22 (0)	8 (2)	3 (4)
	No RT	14 (0)	8 (2)	4 (3)

Supplemental Figure S3. Overall Survival stratified by number of lines of systemic therapy prior to ICB

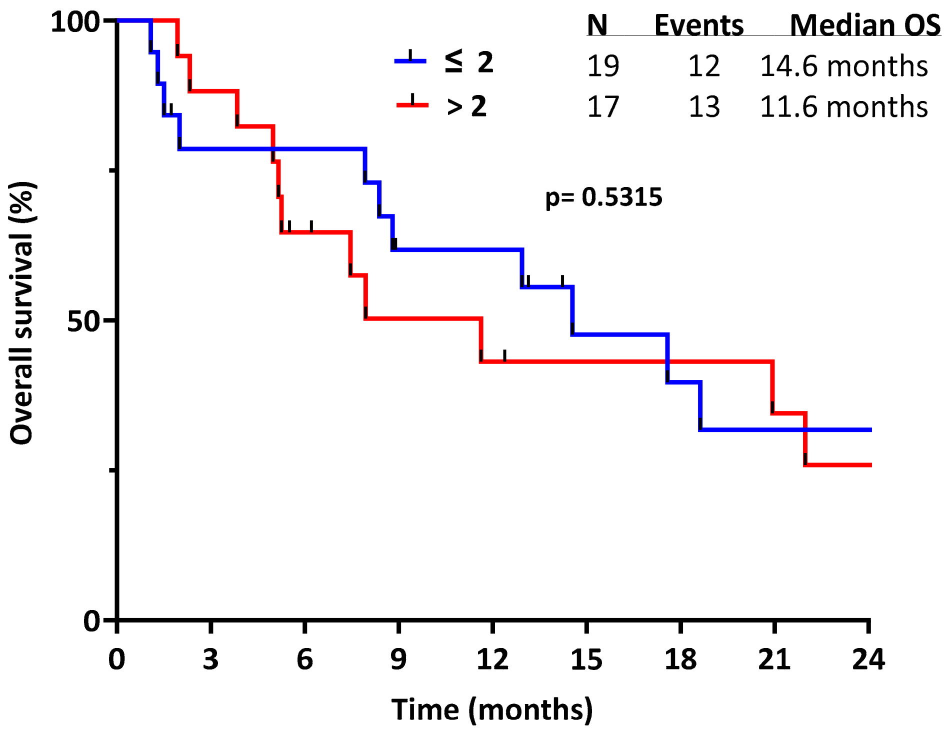

Number at risk (number censored)										
	<u><</u>2	19 (0)	14 (1)	14 (1)	10 (2)	10 (2)	6 (4)	5 (4)	4 (4)	4 (4)
	>2	17 (0)	15 (0)	10 (1)	7 (2)	6 (2)	5 (3)	5 (3)	4 (3)	3 (3)

Supplemental Figure S4. Overall Survival stratified by type of ICB treatment

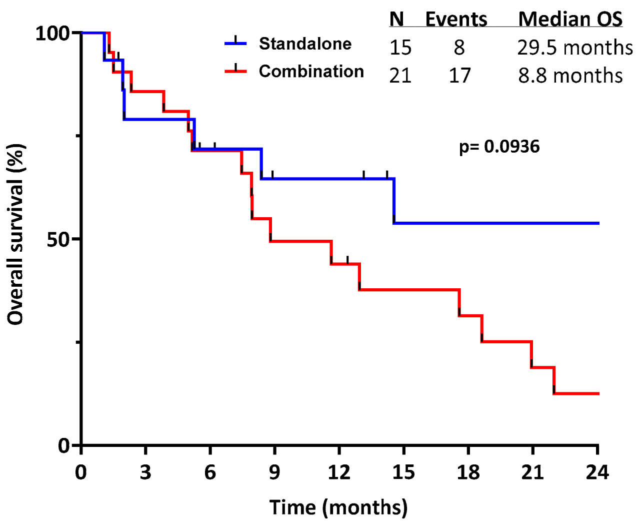

Number at risk (Number censored)										
	Standalone	15 (0)	11 (1)	10 (1)	8 (2)	8 (2)	5 (3)	5 (3)	5 (3)	5 (3)
	Combination	21 (0)	18 (0)	14 (1)	9 (2)	8 (2)	6 (3)	5 (3)	3 (3)	2 (3)
